# Relationship Between Matrix Cracking and Delamination in CFRP Cross-Ply Laminates Subjected to Low Velocity Impact

**DOI:** 10.3390/ma12233990

**Published:** 2019-12-02

**Authors:** Riming Tan, Jifeng Xu, Wei Sun, Zhun Liu, Zhidong Guan, Xia Guo

**Affiliations:** 1 School of Aeronautic Science and Engineering, Beihang University, Beijing 100191, China; 2 Beijing Aeronautical Science and Technology Research Institute, COMAC, Beijing 102211, China; xujifeng@comac.cc; 3 Unmanned Aerial Vehicle Technology Institute, the Third Academy of CASIC, Beijing 100074, China; wsiune@163.com; 4 Tactical Weapons Division, China Academy of Launch Vehicle Technology, Beijing 100076, China; zhunliu@139.com; 5 Beijing Center for Physical and Chemical Analysis, Beijing 100094, China; guoxialihua@163.com

**Keywords:** low-velocity impact, matrix cracking, delamination, extended finite element method, cohesive element

## Abstract

The effect of matrix cracking on the delamination morphology inside carbon fiber reinforced plastics (CFRP) laminates during low-velocity impact (LVI) is an open question. In this paper, the relationship between matrix cracking and delamination is studied by using cross-ply laminates. Several methods, including micrograph, C-scan, and visual inspection, were adopted to characterize the damage after LVI experiments. Based on the experimental results, finite element (FE) models were established to analyze the damage mechanisms. The matrix cracking was predicted by the extended finite element method (XFEM) and the Puck criteria, while the delamination was modeled by cohesive elements. It was revealed that the matrix crack in the bottom ply not only promoted the outward propagation of delamination but also contributed to the narrow delamination beneath the impact location. Multiple matrix cracks occurred in the middle ply. The ones close to the plate center initiated the delamination and prevented large-scale delamination beneath the impact location. For the cracks that were far away, no significant effect on delamination was found. In conclusion, the stress redistribution caused by the crack opening determines the delamination.

## 1. Introduction

Carbon fiber reinforced plastics (CFRP) have been widely applied to aeronautical engineering for several decades due to their high specific strength and stiffness [1]. Notwithstanding, delamination caused by low-velocity impact (LVI) remains a major concern to researchers since it reduces the residual strength considerably.

Although researchers have made numerous efforts in the impact mechanisms study and revealed that the delamination is induced by matrix cracking [2,3,4], the formation of the undelaminated region beneath the impact location is still an open question. According to the opinion of Aymerich [5], the through-thickness compression under the impactor during LVI suppressed the delamination. However, some researchers attributed this phenomenon to the friction between two plies [6,7]. During compression after impact (CAI), the delamination inside a laminate grew into the undelaminated region, leading to the final failure [8,9]. Therefore, it is meaningful to further investigate the reason why the delamination is suppressed.

With the purpose of analyzing the impact mechanisms and predicting the damage, many finite element (FE) models were proposed by researchers. Since the matrix cracking has a close relationship with the delamination initiation, it is necessary to take their interaction into consideration in FE modeling for an accurate prediction in delamination. In some studies, FE models were established based on continuum damage mechanics (CDM). The effect of matrix damage on stress redistribution was considered by modifying the damage evolution behaviors of CDM [10,11]. However, when using CDM, the damage is represented by the material degradation of a traditional FE. Due to the lack of any additional degree of freedom (DOF) to simulate the crack opening inside, the element fails to represent the displacement discontinuity across a matrix crack, leading to inaccurate predictions of the delamination propagation. In several studies, to consider this discontinuity, cohesive elements were not only positioned at interlaminar interfaces to model delamination but also placed in the plies to simulate the matrix cracking [12,13]. Although this method enables the model to simulate the interaction between matrix cracking and delamination, it exhibits some drawbacks, including the requirements of knowing crack paths beforehand and mesh alignment. Moreover, this technique fails to define the intralaminar damage initiation correctly, since not all components of the mechanical constitutive behavior are computed in a cohesive element.

The extended finite element method (XFEM) proposed by Belyschko and Blacks [14] is an attractive method to model cracking. It is suitable to represent the displacement discontinuity and stress concentration near the crack tip. Combined with cohesive zone models (CZM), it is capable of modeling cohesive crack propagation without remeshing or defining any crack path in FE models. Proved by several studies [15,16,17], XFEM can be an ideal technique in matrix cracking modeling if an appropriate failure theory is employed. Compared to other failure theories of composite materials, for example, the Tsai-Wu criterion and Hashin criteria, the Puck criteria [18] have obvious advantages in the matrix failure prediction since they can describe the relationship between the matrix crack direction and stress state. The combination of XFEM and the Puck criteria is expected to fully explain the interaction between the matrix cracking and stress distribution.

The main goal of this paper is to figure out the influence of matrix cracking on delamination morphology during LVI, especially the undelaminated region beneath the impact location. An FE modeling strategy based on XFEM, the Puck criteria, and cohesive elements is proposed to predict matrix cracking and delamination accurately. The simulations are verified by the experimental results. Cross-ply laminates were utilized in this paper due to their simple damage mechanisms. Although many researchers studied the damage mechanisms by applying cross-ply laminates [2,3,19,20,21], the impact experiment results in these studies are hard to reproduce since their experiments were not standardized. In this paper, the cross-ply laminates were tested according to ASTM D7136 [22]. How the stress redistribution caused by the matrix cracking determines the undelaminated region is discussed, which has not been studied by other researchers to the best of the authors’ knowledge.

## 2. Materials and Experiments

Cross-ply laminates tested in this paper were manufactured from carbon fiber reinforced epoxy resin composite IMA/M21 prepregs by an automatic tape-laying technique. The prepregs were supplied by Hexcel Corporation (Stamford, Connecticut, USA). The stacking sequence was designed to be ${\left[0_{3}/90_{3}\right]}_{S}$. All laminates were cured in an autoclave with steel plate mold. A vacuum bag was used to apply a full vacuum. The heat-up rate from room temperature was controlled between 1–2 °C/min. The temperature was held at 180 °C for 120 min under 7 bar gauge autoclave pressure before cooling down to room temperature with a slow rate (2–5 °C/min). After curing, the thickness was 2.16 mm on average. To satisfy the requirement of the standard, the nominal length and width were 150 and 100 mm, respectively.

**Figure 1:**
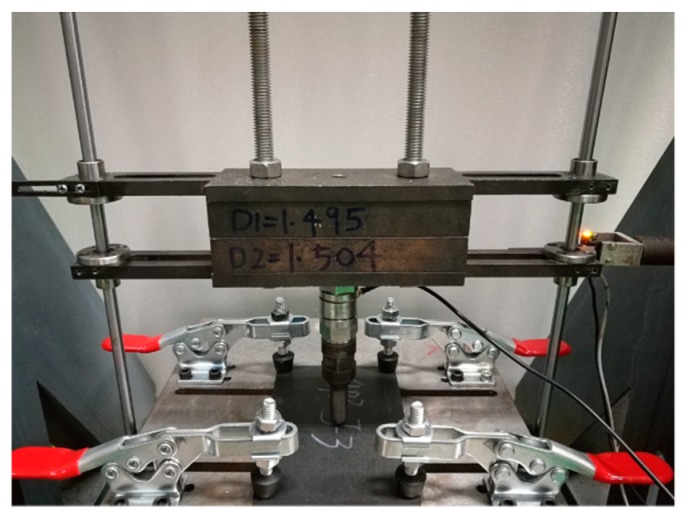
Boundary condition of the impact experiments.

Impact damage was introduced to the center of each specimen at room temperature by applying a drop-weight impact instrument, which was designed according to ASTM D7136. The impactor used in this test consisted of a hemispherical nose with a diameter of 16 mm. The total weight of the impactor was 5.495 kg. The specimens were placed on a fixture base with a 125 × 75 mm cut-out. Meanwhile, the corners of the specimens were restrained by four clamps. The boundary condition of the impact experiments is presented in Figure 1. In this research, the impact energy levels were decided to be 4, 8, and 12 J to induce damage at different degrees inside the specimens. Correspondingly, the specimens were divided into three groups, which were labeled as Group A, B, and C. Each group contained four specimens.

After the impact experiments, the impact dent depth was measured with a Syntek micrometer gauge (Syntek Corporation, Huzhou, China) immediately. The impact damage was inspected by visual inspection and ultrasonic C-scan. A UC-120 C-scanner equipped with a 10 MHz ultrasonic probe was used. Both the C-scanner and probe were products of Physical Acoustics Corporation (Princeton, NJ, USA). To characterize internal impact damage, one specimen in each group was cut along the longitudinal direction. The internal damage was observed with an MV-GED500M industrial camera manufactured by Shenzhen Mindvision Technology CO., LTD (Shenzhen, China).

## 3. Finite Element Modeling Strategy

FE models were established in the commercial FE software ABAQUS 6.13 to simulate the impact processes using subroutines UDMGINI and UMAT. As shown in Section 4.1, no fiber breakage occurred in the experiments; thus, only matrix cracking and delamination were taken into consideration. The matrix cracking initiation was predicted by the Puck criteria, while the cracking opening was governed by XFEM-based CZM. Delamination was modeled by cohesive elements.

### 3.1. Intralaminar Damage Model

#### 3.1.1. Puck Criteria

In this study, the Puck criteria proposed by Puck and Schürmann [18] were employed for the matrix cracking prediction. Puck criteria have been widely used [23,24,25] since they can predict the crack initiation and its angle successfully. The fracture angle is important in LVI simulations. It influences the stressing significantly when the damage initiates. The stress exposure factor $f_{E}$ of the Puck criteria for matrix cracking prediction is expressed as [26]: for $\sigma_{n}\left(\theta \right)\geq 0$:

(1) $$f_{E}\left(\theta \right)=\sqrt{{\left[\left(\frac{1}{R_{⊥}^{\left(+\right)A}}-\frac{p_{⊥\psi }^{\left(+\right)}}{R_{⊥\psi }^{A}}\right)\sigma_{n}\left(\theta \right)\right]}^{2}+{\left(\frac{\tau_{nt}\left(\theta \right)}{R_{⊥⊥}^{A}}\right)}^{2}+{\left(\frac{\tau_{nl}\left(\theta \right)}{R_{⊥||}^{A}}\right)}^{2}}+\frac{p_{⊥\psi }^{\left(+\right)}}{R_{⊥\psi }^{A}}\sigma_{n}\left(\theta \right),$$

for $\sigma_{n}\left(\theta \right)<0$:

(2) $$f_{E}\left(\theta \right)=\sqrt{{\left[\frac{p_{⊥\psi }^{\left(-\right)}}{R_{⊥\psi }^{A}}\sigma_{n}\left(\theta \right)\right]}^{2}+{\left(\frac{\tau_{nt}\left(\theta \right)}{R_{⊥⊥}^{A}}\right)}^{2}+{\left(\frac{\tau_{nl}\left(\theta \right)}{R_{⊥||}^{A}}\right)}^{2}}+\frac{p_{⊥\psi }^{\left(-\right)}}{R_{⊥\psi }^{A}}\sigma_{n}\left(\theta \right),$$

with

(3) $$\{\begin{array}{c} \frac{p_{⊥\psi }^{\left(+\right)}}{R_{⊥\psi }^{A}}=\frac{p_{⊥⊥}^{\left(+\right)}}{R_{⊥⊥}^{A}}\cos^{2}\psi +\frac{p_{⊥||}^{\left(+\right)}}{R_{⊥||}^{A}}\sin^{2}\psi \\ \frac{p_{⊥\psi }^{\left(-\right)}}{R_{⊥\psi }^{A}}=\frac{p_{⊥⊥}^{\left(-\right)}}{R_{⊥⊥}^{A}}\cos^{2}\psi +\frac{p_{⊥||}^{\left(-\right)}}{R_{⊥||}^{A}}\sin^{2}\psi \end{array}\;\mathrm{and}\;\{\begin{array}{c} \cos^{2}\psi =\frac{\tau_{nt}^{2}}{\tau_{nt}^{2}+\tau_{nl}^{2}}\\ \sin^{2}\psi =\frac{\tau_{nl}^{2}}{\tau_{nt}^{2}+\tau_{nl}^{2}} \end{array}\;\mathrm{for}\;\tau_{nt}^{2}+\tau_{nl}^{2}>0,$$

(4) $$\frac{p_{⊥\psi }^{\left(+\right)}}{R_{⊥\psi }^{A}}=\frac{p_{⊥\psi }^{\left(-\right)}}{R_{⊥\psi }^{A}}=0\;\mathrm{for}\;\tau_{nt}^{2}+\tau_{nl}^{2}=0$$

where $p_{⊥⊥}^{\left(+\right)}$, $p_{⊥⊥}^{\left(-\right)}$, $p_{⊥||}^{\left(+\right)}$, and $p_{⊥||}^{\left(-\right)}$ are inclination parameters and their values were recommended by Puck [26,27]; $R_{⊥⊥}^{A}$, $R_{⊥||}^{A}$, and $R_{⊥}^{\left(+\right)A}$ are fracture resistances of the action plane against the fracture, and equal $Y_{C}/2\left(1+p_{⊥⊥}^{\left(-\right)}\right)$, the in-plane shear strength $S_{12}$, and transverse tensile strength $Y_{T}$ respectively [18]; $\sigma_{n}\left(\theta \right)$, $\tau_{nt}\left(\theta \right)$, and $\tau_{nl}\left(\theta \right)$ are stress components on an arbitrary fiber parallel action plane with an inclination angle θ and obtained by the following equations:

(5) $$\begin{array}{l} \sigma_{n}=\sigma_{22}\cos^{2}\theta +\sigma_{33}\sin^{2}\theta +2\tau_{23}\cos\theta \sin\theta \\ \tau_{nt}=\left(\sigma_{33}-\sigma_{22}\right)\sin\theta \cos\theta +\tau_{23}\left(\cos^{2}\theta -\sin^{2}\theta \right)\\ \tau_{nl}=\tau_{13}\sin\theta +\tau_{12}\cos\theta \end{array}$$

To determine the angle $\theta_{fp}$ of the potential fracture plane efficiently, an algorithm [28] was proposed by modifying the ones established by Wiegand [29] and Schirmaier [30]. Four steps are conducted: (1) calculate N+1 supporting points of the $f_{E}-\theta$ curve at intervals of 180°/N; (2) localize the subranges containing a local maximum; (3) determine the local maxima by inverse parabolic interpolation (IPI) [29]; (4) compare the local maxima to find the global maximum $f_{E}\left(\theta_{fp}\right)$ and its corresponding angle $\theta_{fp}$. Considering the fact that the minimum distance between two local maxima is greater than 25° [30], the numerical error of this algorithm is acceptably small if N=20. When $f_{E}\left(\theta_{fp}\right)$ reaches 1, a matrix crack occurs.

#### 3.1.2. XFEM in ABAQUS

XFEM framework embedded in ABAQUS was used. In ABAQUS, cohesive segments method and phantom nodes are adopted when modeling the moving cracks. Before the crack initiation, the phantom nodes are superposed on the corresponding real nodes. When an element is cut by a crack, DOFs of phantom nodes are independent of those of real nodes. Consequently, the element is cut into two segments. The separation between these two segments leads to the stress discontinuity in a cracked region. Such separation is governed by the following traction-separation cohesive behavior:

(6) $$\begin{array}{l} t_{n}=\{\begin{array}{c} \left(1-D\right)T_{n}=\left(1-D\right)K_{nn}\delta_{n},\;T_{n}\geq 0\\ T_{n}=K_{nn}\delta_{n},\;T_{n}<0 \end{array}\\ t_{s}=\left(1-D\right)T_{s}=\left(1-D\right)K_{ss}\delta_{s}\\ t_{t}=\left(1-D\right)T_{t}=\left(1-D\right)K_{tt}\delta_{t} \end{array}$$

where D is the damage variable (STATUSXFEM); $\delta_{n}$, $\delta_{s}$, and $\delta_{t}$ are the separations in the normal and two shear directions; $K_{nn}$, $K_{ss}$, and $K_{tt}$ are the corresponding stiffness components, and calculated by the material properties of the element.

The critical energy release rate $G_{c}$ of a mixed-mode XFEM cohesive crack was determined by the Benzeggagh–Kenane (B-K) law model [31]:

(7) $$G_{c}=G_{Ic}+\left(G_{IIc}-G_{Ic}\right){\left(\frac{G_{II}+G_{III}}{G_{I}+G_{II}+G_{III}}\right)}^{\eta }$$

where $G_{Ic}$ and $G_{IIc}$ are the critical energy release rates of Mode I and II, respectively; $G_{I}$, $G_{II}$, and $G_{III}$ are the energy release rates of the pure modes; η is a material parameter. η=1.45 was used [32]. When the energy release rate is greater than $G_{c}$, the crack is fully opened, and the tractions between segments reduce to zero.

To enhance the numerical convergence, the viscous regularization technique with a small viscosity coefficient (0.0001) was adopted [33]. In a typical simulation in this paper, the energy dissipated by viscous regularization was about 3.5% of the total energy, proving that the viscosity coefficient was small enough.

#### 3.1.3. Nonlinear Shear Behavior

It has been revealed by many researchers that the transverse/parallel shear behavior of composites exhibits high nonlinearity [34,35,36]. When a ply is under the shear loading, the matrix between fibers is under local tensile stress due to the Poisson ratio mismatch of the fiber and matrix. Hence, the micro-cracking forms in the matrix and leads to the nonlinearity [37,38]. For the cross-ply laminates tested in this paper, high-level shear strain $\gamma_{13}$ occurred near the impact location during LVI. The widely used Ramberg–Osgood equation with three parameters was adopted to describe the nonlinear $\tau_{13}-\gamma_{13}$ behavior [36,39,40]:

(8) $$\tau_{13}=\frac{G_{13}^{0}\gamma_{13}}{{\left(1+{\left(\frac{G_{13}^{0}\gamma_{13}}{\tau_{b}}\right)}^{n}\right)}^{\frac{1}{n}}}$$

where $G_{13}^{0}$ is the initial shear modulus, $\tau_{b}$ is the asymptotic stress level, which is assumed to be equal to $S_{12}$, and n is the shape parameter for the curve. In this paper, $G_{13}^{0}$, $\tau_{b}$, and n were 4.20 GPa, 105 MPa, and 2.0 respectively.

### 3.2. Interlaminar Damage Model

The constitutive response of cohesive elements was also described by the traction–separation relationship (see Equation (6)), indicating that only three stress components ($\sigma_{33}$, $\tau_{13}$, and $\tau_{23}$) were computed. The stiffness components $K_{nn}$, $K_{ss}$, and $K_{tt}$ of cohesive elements are assumed to be large numbers [41]. In this study, $10^{6}N/mm^{3}$ proposed by Camanho [42] was used. The delamination initiation and evolution were modeled with a quadratic nominal stress criterion and the B-K law (see Equation (7)), respectively. The quadratic nominal stress criterion can be represented as:

(9) $${\left(\frac{\langle \sigma_{33}\rangle }{N}\right)}^{2}+{\left(\frac{\tau_{13}}{S}\right)}^{2}+{\left(\frac{\tau_{23}}{S}\right)}^{2}=1$$

where N and S are the normal and shear strengths of the interface; the symbol 〈〉 is the Macaulay bracket.

### 3.3. Finite Element Modeling

**Figure 2:**
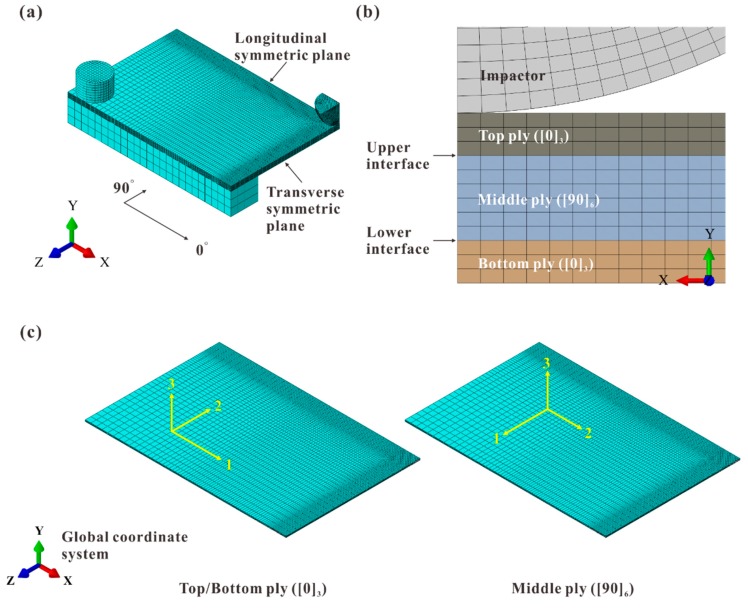
Finite element (FE) model of the cross-ply laminate: (**a**) isometric view; (**b**) details of side view; (**c**) ply coordinate systems (in yellow) of each ply.

A quarter of the cross-ply laminate was built in FE models due to the symmetry (see Figure 2). As to verify the validity of the modeling method, all impact cases in the experiments were analyzed by ABAQUS/Implicit solver. The FE models corresponding to the aforementioned impact energy levels were named as Model A, B, and C. The ply coordinate systems were presented as the yellow ones in Figure 2c. It should be mentioned that the stress components in the ply coordinate systems were used in the following discussion about the intralaminar stress distributions.

To model the boundary conditions, a base and clamp were established. Both of them were constrained as rigid bodies; meanwhile, all DOFs were fixed. For the purpose of improving the convergence, the impactor was modeled as a deformable part and meshed with linear solid elements with reduced integration (C3D8R). Symmetric boundary conditions were applied to the symmetric planes. Interactions between parts were all defined as surface-to-surface contact. The contact between plies after delamination was also taken into consideration. Friction coefficients of all contact pairs were set as 0.3. Composite plies were modeled with linear solid elements (C3D8) to improve the crack calculation accuracy. Two interlaminar interfaces were simulated with cohesive elements (COH3D8). For composite plies, a fine mesh with 0.3 mm for the in-plane size was used near the impact location. The element size increased to 2 mm at the edges of the laminate. To improve the accuracy of modeling delamination shape, the size of cohesive elements was determined to be 0.2 mm in the prospective delaminated regions. These mesh sizes satisfied the mesh convergence according to the preliminary simulations. The total number of elements was 95,178.

The material properties were listed in Table 1. For the interlaminar interfaces, the typical $G_{Ic}$ and $G_{IIc}$ of carbon fiber/epoxy resin composites reported by Caminero [43] were adopted. The ones of matrix cracking were assumed to be the same. In the preliminary simulations, the delamination sizes and length of the crack in the bottom ply were considerably smaller than the experimental ones. As revealed in the simulations, this crack was caused by the transverse tensile stress $\sigma_{22}$, and its insufficient propagation restricted the delamination. The experimental study of Mortell [44] indicated that during bending, the transverse stress for matrix crack initiation in the outmost ply was lower than $Y_{T}$ of unidirectional plies, and decreased when the ply thickness increased, which was the so-called “in-situ” effect [45]. This was believed to be related to the residual thermal stress [45] and stress concentration due to the non-uniform distribution of carbon fiber [46]. In addition, the defects which reside on the carbon fiber surface also contribute to this phenomenon, since they reduce the fiber/matrix interfacial strength [47,48] and further lead to the matrix cracking [45,46]. In this research, $Y_{T}$ of the bottom ply was decreased to 20 MPa.

According to the study of Chen [49], a matrix crack can introduce damage to all integration points of its adjacent cohesive element, since this element is unable to be partitioned by a crack. The damaged element further cause damage to all adjacent elements at the interface. In this study, a shear crack induced such artificial damage between the crack and impact location. This phenomenon was inevitable, no matter how small the element size was used [6]. However, for the elements in this region, the increases in their damage variables (SDEG) were inhibited by the stress release as detailed in Section 4.2.2, and these elements were still capable of bearing the stress (at least 20 MPa), even though their SDEG were larger than 0.9. Therefore, only the cohesive elements whose SDEG became 1.0 were considered as failure [50] and regarded as the delaminated region.

## 4. Results and Discussion

In this section, the experimental results, mainly the damage modes and their sizes, are presented firstly. During the experiments, these damage sizes, including the delamination sizes and angles of matrix crack, were measured by the image analysis of C-scan data and micrograph. Later, the damage modes and sizes are used for qualitative and quantitative verification of the modeling strategy. As revealed by the Puck criteria, the angle of a matrix crack is determined uniquely by the stress state. Hence, the numerical results of crack angles can be used for the verification of the stress calculation. After the simulations are verified, the stress distributions and damage formation in the simulations are used for the damage mechanisms analysis with the aid of the failure theories mentioned in Section 3.

### 4.1. Experimental Results

**Figure 3:**
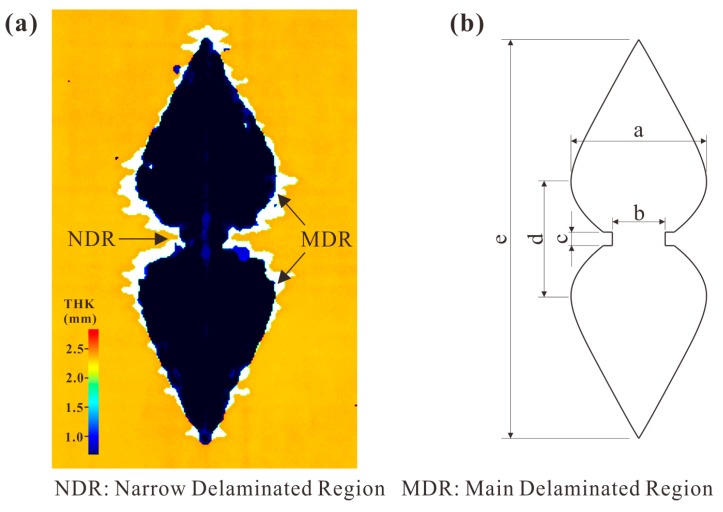
Delamination: (**a**) the one detected by the C-scan; (**b**) the idealized shape.

As revealed by the C-scan and micrograph of the internal damage, delamination only existed at the lower interface. The typical delamination detected by C-scan from the lower surface is displayed in Figure 3a. The delamination was in a “peanut shape” [5]. The main delaminated region (MDR) consisted of two lobes. They were almost symmetric regardless of the dynamic effect. A narrow delaminated region (NDR) beneath the impact location connected these two lobes. Each lobe was sharp at the tip and shrank near the impact location.

**Figure 4:**
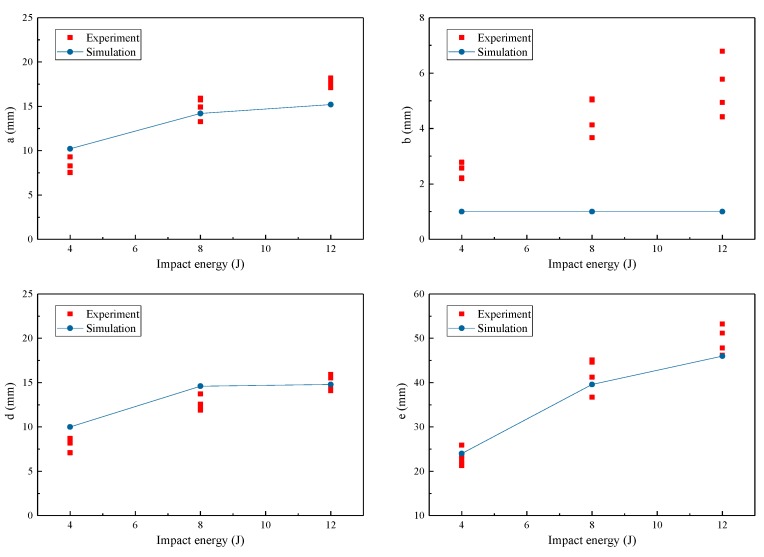
Delamination sizes of all specimens and numerical results.

For each specimen, the outline of the delamination was idealized, as in Figure 3b. The sizes of the simplified shape were measured by the positional data obtained from C-scanner. As detailed in Table 2, “c” almost remained as a constant, while the other sizes increased with the energy monotonically. Figure 4 presents the changes in the delamination sizes for all specimens and simulations. Since “c” was independent of the impact energy, the change trends of the other sizes are shown. The values of “a” and “d” were below 10 mm for the impact energy of 4 J, but increased to about 15 mm for 12 J. As for “e”, it increased with the impact energy in a large scale, which was larger than 30 mm when energy increased from 4 to 12 J, but the growth of “b” was relatively small.

**Figure 5:**
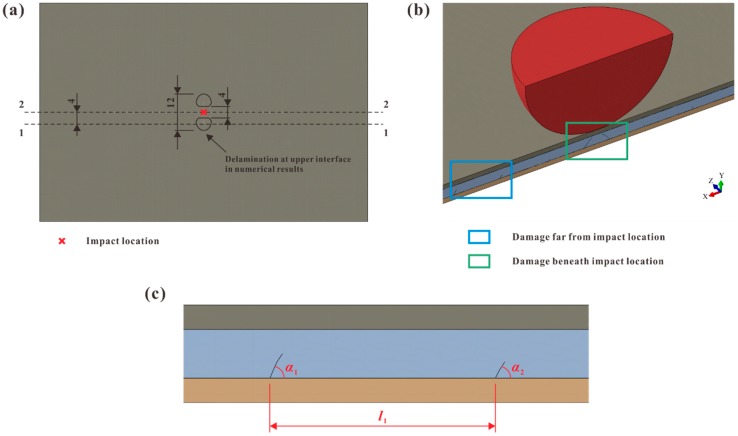
Details of micrograph approach: (**a**) locations of the sections for observation (unit: mm); (**b**) locations for damage photograph on Section 2-2 (in the blue and green boxes); (**c**) measurement of the crack angles and distances.

A micrograph was used for revealing the internal damage at different locations (see Figure 5a,b). In the numerical results, delamination occurred at the upper interface and was close to the impact location (see black solid lines in Figure 5a). To verify whether such delamination existed in the experiments, the damage on Section 1-1 of a specimen was studied. As shown in Figure 5a, Section 1-1 was parallel to Section 2-2, which was the longitudinal symmetric plane. Their distance was determined by the sizes of the aforementioned delamination in the simulations. For each group, the damage on Section 2-2 was recorded. The angles and distances of the matrix crack were measured as in Figure 5c.

**Figure 6:**
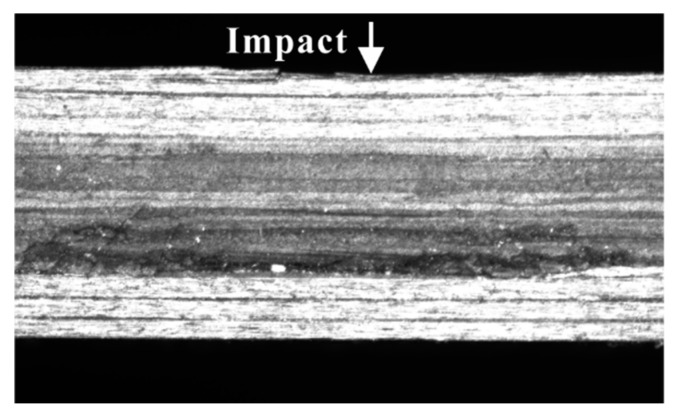
Damage observed by micrograph on Section 1-1 for 12 J.

**Figure 7:**
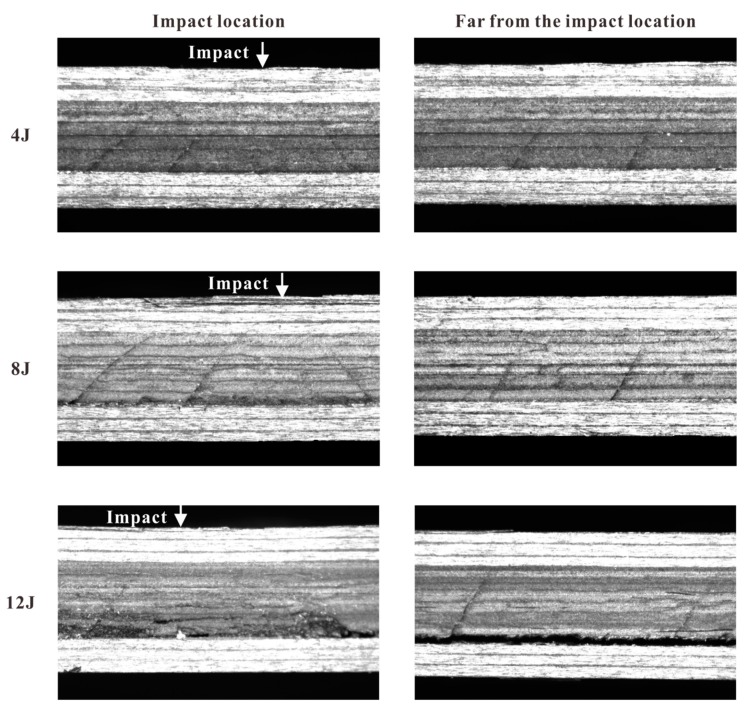
Damage observed by micrograph on Section 2-2 for three impact energy levels.

Figure 6 indicates that no delamination occurred at the upper interface. LVI caused only matrix damage and delamination at the lower interface for all impact energy levels in this research (see Figure 7). 

Delamination extended along the longitudinal direction with a long distance, including the region beneath the impact location. Due to the permanent deformation of the bottom ply after delamination and debris between the delaminated surfaces [12], the gap between the bottom ply and middle one formed in the delaminated region of the lower interface. This gap became evident when the impact energy was high enough, as shown in the subgraphs for 12 J in Figure 7.

Multiple matrix cracks started at the lower interface and propagated towards the impact location. All of them were confined in the middle ply. When the impact energy increased to 12 J, matrix crushed in the middle ply beneath the impact location. Although the numerical results showed that both the tensile transverse stress $\sigma_{22}$ and out-of-plane shear stress $\tau_{23}$ triggered off these cracks, they were still named as shear cracks for simplicity, similarly to Bouvet [12].

For each impact case, the crack that was far from the impact location usually propagated with a larger inclination compared to the crack close to the impact location. The angles of the farthest crack for three impact energy levels (4, 8, and 12 J) were 58.0°, 61.2°, and 64.6°, respectively. As for the closet crack, its angles were 53.6°, 51.7°, and 50.7° for the three impact cases. Probably related to the randomness of defects, the crack spacing varied from 2 to 7 mm without regularity.

**Figure 8:**
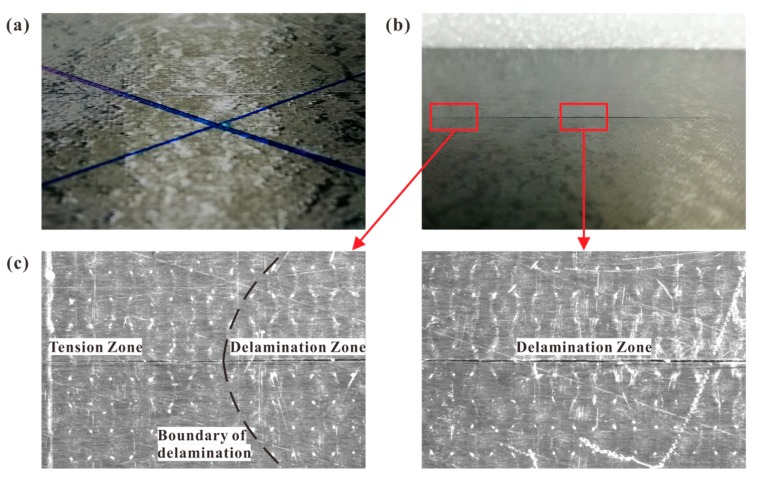
(**a**) Impact dent on the upper surface; (**b**) tensile crack on the lower interface; (**c**) details of tensile crack observed by micrograph.

The typical external impact damage was presented in Figure 8. No severe damage, for example, fiber breakage, was caused by LVI on either surface. On the upper surface, only a shallow dent was found (see Figure 8a). The average dent depths were 0.054, 0.125, and 0.228 mm for Group A, B, and C, respectively. As for the lower interface, only a long tensile crack was found (see Figure 8b). This crack consisted of two zones: Tension Zone at the tips and Delamination Zone beneath the impact location (see Figure 8c). They were named because the initiation of the former was only related to the transverse tensile stress $\sigma_{22}$; however, the latter also had a close relationship with the delamination. The most significant difference between these two zones was that the crack opening distance (COD) of the Delamination Zone was larger than that of the Tension Zone. It can be explained as follows: during the unloading stage of LVI, the crack closure of the Delamination Zone was hindered by the debris [12] and permanent deformation of the bottom ply after delamination. Additionally, the Tension Zone constituted at least half of the total length of the crack. Meanwhile, the length of the Delamination Zone was almost identical to the delamination length. For example, they were 49.20 and 49.69 mm, respectively, for a specimen in Group C.

Since the damage modes and their symmetry remained unchanged in all impact cases in the experiments, the modeling strategy proposed in this paper was reasonable.

### 4.2. Numerical Results

#### 4.2.1. Damage Modes and Sizes Verification

The numerical results verified the ability of the modeling strategy to capture the main damage modes and their sizes, i.e., the delamination at the lower interface and matrix cracks. Although isolated artificial delamination occurred near the NDR, it was neglected in the discussions about damage mechanisms, since it was only induced by the limitations of cohesive elements and had no influence on the damage mechanisms.

**Figure 9:**
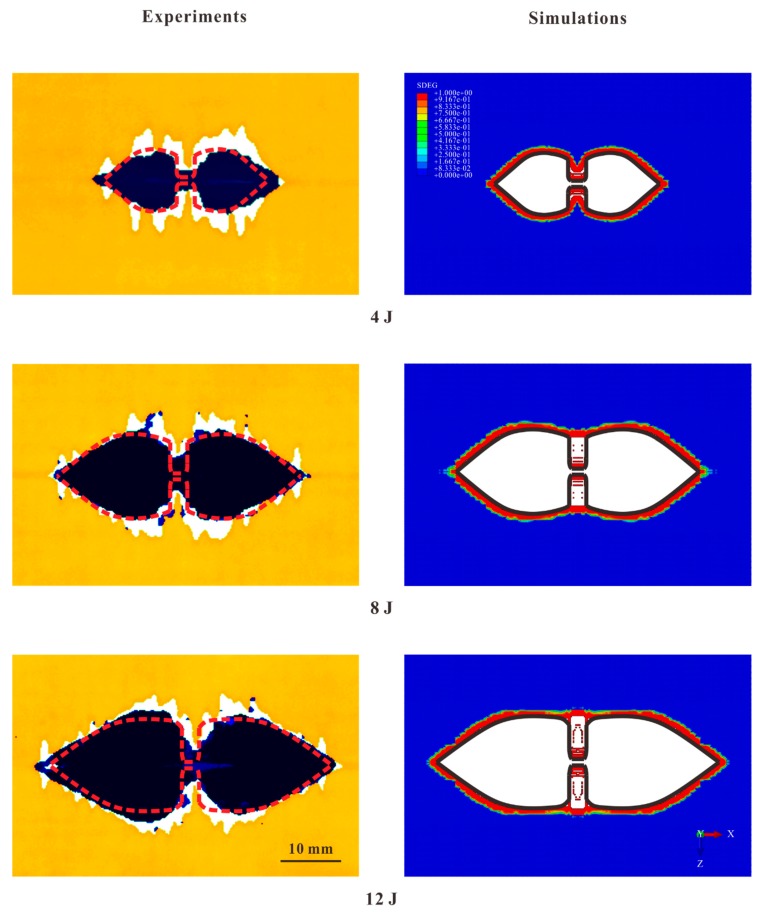
Comparison between experimental and numerical results of delamination for three impact energy levels. The outlines of the numerical results of the delamination are represented by the thick lines (the red ones in the left figures and the black ones in the right figures).

The comparison in Figure 9 indicates that the delamination shapes in the numerical results were in good agreement with the experimental ones for all impact energy levels. In each model, the delamination was in the peanut shape in which the major axis lay along the longitudinal direction. The shape was composed of two lobes (MDR) and a narrow tape (NDR) that connected these lobes.

The FE modeling strategy could also predict the delamination sizes successfully since the differences in most sizes between the experimental and numerical results were acceptably small (see Table 2 and Figure 4). In the simulations, “a”, “d”, and “e” also increased with the impact energy. For “c”, it remained as a constant regardless of the impact energy. Only for “b”, the simulation results and experimental ones were not in good agreement. This phenomenon was believed to be related to the insufficient COD of the tensile crack. During the impact, the COD was smaller than 0.6 mm, which limited the deformation of the cohesive elements at the edges of the NDR, including the normal tension and shear deformation in the Y-Z plane. As revealed in Section 4.2.2, the failure of a cohesive element in this region was mainly caused by the deformation in these two directions. Therefore, the widening of the NDR of the bottom interface lagged behind the growth of other delamination sizes.

**Figure 10:**
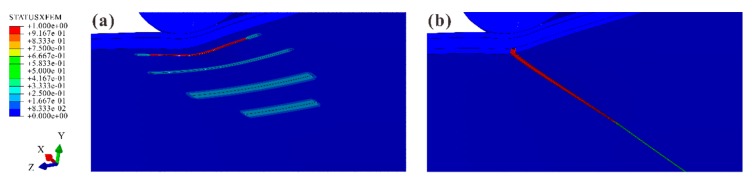
Matrix cracks: (**a**) in the middle ply; (**b**) in the bottom ply.

**Figure 11:**
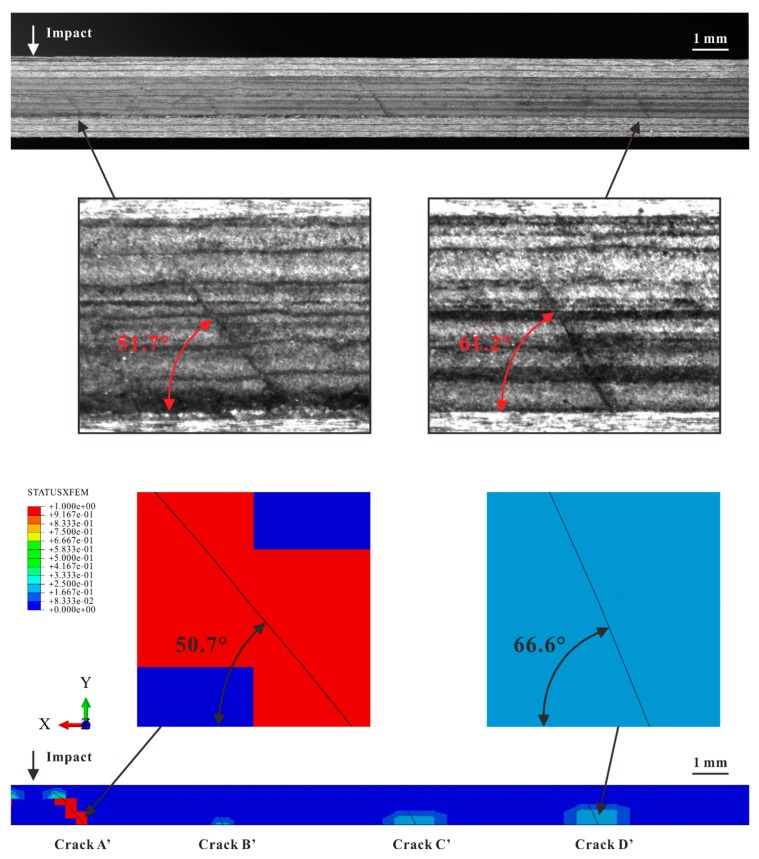
Comparison between experimental and numerical results of shear cracks in the middle ply for 8 J.

In FE models, two kinds of matrix cracks were also observed, including multiple shear cracks in the middle ply and a tensile crack in the bottom ply (see Figure 10). The comparison in Figure 11 verifies that the numbers and angles of the shear cracks in the numerical results were consistent with the experimental ones. The case with an impact energy of 8 J is selected as an illustrative example for the discussion about the shear cracks.

Similar to the experimental results, most shear cracks propagated upwards linearly with different inclines and stopped in the middle ply. For simplification, these cracks are hereinafter referred to as Crack A’, B’, C’, and D’ according to their locations. Crack A’ was the one closest to the impact location. As mentioned in Section 4.1, in the experiments, the inclination angles increased with the distance to the impact location in general. This tendency was also captured by the simulations. For instance, the experimental angles of Crack A’ and D’ were 51.7° and 61.2°, respectively, while the corresponding angles in Model B were 50.7° and 66.6°. It indicates that the models predicted the stress distributions accurately since the crack angles were dependent on the stress components ratio $\sigma_{22}:\tau_{23}$ according to the Puck criteria for matrix cracking initiation. The ratios for Crack A’ and D’ were 0.45 and 2.41. When the impact energy increased, more shear cracks occurred in both experimental and numerical results. The numbers of shear cracks in the specimens for micrograph were 7 (4 J), 8 (8 J), and 11 (12 J). The numbers in the corresponding FE models were 6, 8, and 10. Like the experimental results, the impact resulted in the tensile crack with a long cohesive zone. Due to the downward movement of the impactor, the crack was fully opened beneath the impact location. The cohesive crack tip and fully opened part corresponded to the Tension Zone and Delamination Zone in Figure 8, respectively.

Since the models were capable of simulating the main damage modes and their trends, it was reasonable to analyze the damage mechanisms based on the numerical results.

#### 4.2.2. Damage Mechanisms Analysis

**Figure 12:**
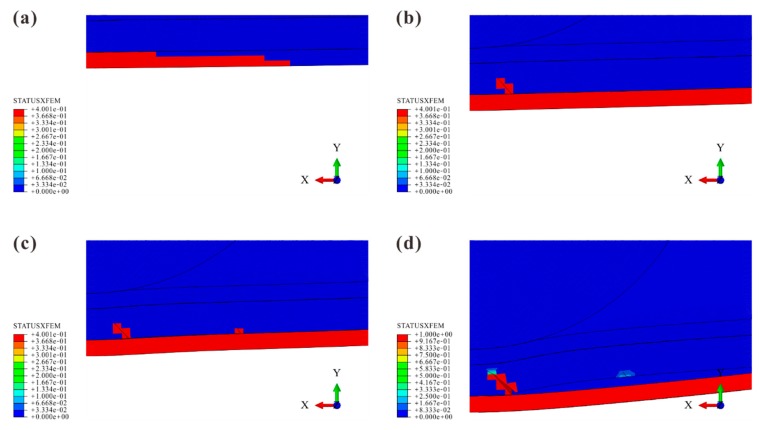
Matrix cracking: (**a**) tensile crack in the bottom ply (680 μs); (**b**) propagation of Crack A’ (1568 μs); (**c**) propagation of Crack B’ (2011 μs); (**d**) almost fully opening of Crack A’ (3567 μs).

**Figure 13:**
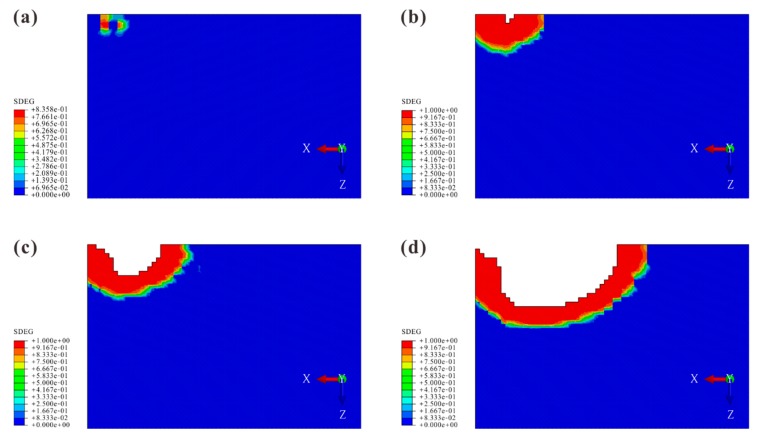
Delamination: (**a**) damage caused by Crack A’ (1528 μs); (**b**) delamination initiation (1652 μs); (**c**) narrow delaminated region (NDR) occurrence (1970 μs); (**d**) delamination propagation (2389 μs).

The damage processes for all models were similar. Hence, the process of Model B is presented as an illustrative example. The propagation of matrix cracking and delamination is shown in Figure 12 and Figure 13 to explore which cracks dominated the delamination.

The tensile crack in the bottom ply was the first damage. Induced by the tensile $\sigma_{22}$, it started in the lowest element beneath the impact location and propagated outwards along the longitudinal direction. Due to the in-situ effect, $Y_{T}$ of the outmost ply was low. Therefore, small out-of-plane deformation of the laminate led to a large scale of the Tension Zone. Afterward, caused by $\sigma_{22}$ and $\tau_{23}$, Crack A’ occurred at the bottom of the middle ply. It propagated along the transverse direction and upwards. Cohesive elements beneath Crack A’ became damaged due to the stress discontinuity. Later, the delamination occurred and propagated outwards from Crack A’. When the out-of-plane deformation became large enough, the delamination also propagated towards the impact location, initiating the NDR. However, the delamination was suppressed in the region between Crack A’ and impact location except for the NDR. The downward movement of the impactor not only induced more shear cracks but also enlarged CODs of Crack A’ and the tensile crack. For the Delamination Zone of the tensile crack, its outward propagation and the increase in its COD forced the delamination to propagate simultaneously. Although the appearance of the other cracks (Crack B’, C’, and D’) resulted in damage to their corresponding cohesive elements, no obvious influence on the delamination was observed.

**Figure 14:**
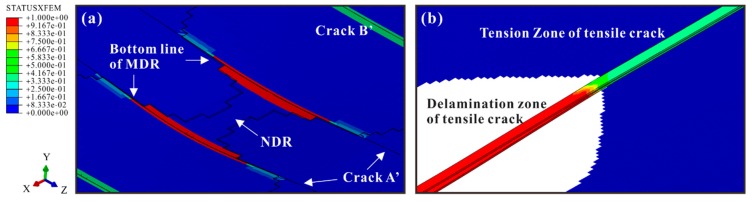
Interactions between matrix cracks and delamination: (**a**) shear cracks; (**b**) tensile crack.

Consequently, as illustrated in Figure 14, the delamination was constrained by Crack A’ and the Delamination Zone. The former became the bottom line of the MDR. The latter restrained the length of the MDR and contributed to the widening of the NDR. The effects of these cracks on the delamination were detailed as follows.

For the tensile crack, the stress discontinuity induced damage to the cohesive elements at the lower interface. As the impactor moved downwards, the stress jump caused by COD kept increasing and further encouraged the delamination. The crack opening also degraded the bending stiffness of the laminates, which, in turn, facilitated the crack growth. Hence, the delamination propagation was accelerated. At the bottom line of the MDR, delamination propagated towards the impact location was dominated by the shear deformation in the X-Y plane. For a cohesive element in this region, the shear strain (2.0%) was much larger than the normal strain (0.2%) before its deletion. Beneath the impact location, the crack opening induced the large deformation at the lower interface, including the normal deformation and shear deformation in the Y-Z plane. The deformation initiated the narrow delamination in this region. These can be proved by the failure strain of a cohesive element in this region: 2.1% (the normal tensile strain) and 1.5% (shear strain in the Y-Z plane).

**Figure 15:**
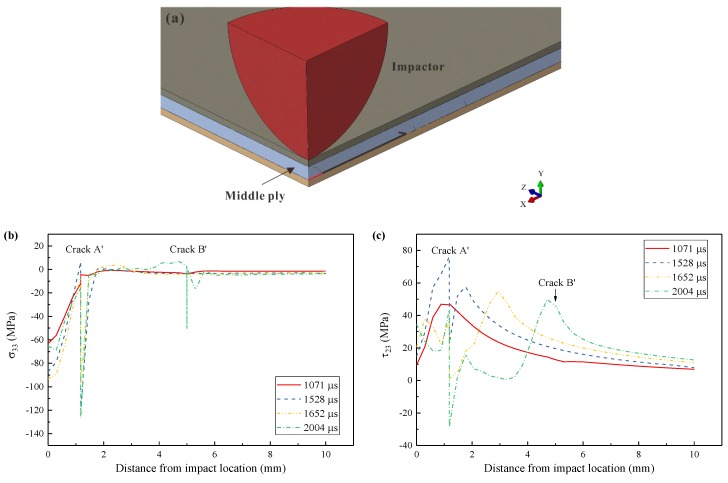
Stress distribution at the bottom of the middle ply along the longitudinal direction: (**a**) the path for stress distribution study (the red line represents the Inner Zone, the black line represents the Outer Zone); (**b**) $\sigma_{33}$; (c) $\tau_{23}$.

The stress distributions of a path were studied to evaluate the effects of Crack A’. As illustrated in Figure 15a, this path was at the bottom of the middle ply in the longitudinal symmetric plane. It was divided into Inner Zone and Outer Zone by Crack A’. The distributions of $\sigma_{33}$ and $\tau_{23}$ at different moments are compared in Figure 15b,c since they were possibly the major influencing factors in delamination propagation.

$\sigma_{33}$ reached its maximum right beneath the impact location and decreased gradually with the distance. It almost decreased to zero where Crack A occurred. Shortly after the initiation of Crack A’ (1071 μs), this crack induced a small jump of $\sigma_{33}$. As COD of Crack A’ kept increasing, the stress discontinuity became pronounced. Meanwhile, the compression stress $\sigma_{33}$ became larger in the Inner Zone. However, $\sigma_{33}$ remained almost zero in the Outer Zone. Even after the delamination occurred (1652 μs), $\sigma_{33}$ still remained at the low level in the Outer Zone. After the occurrence of Crack B’ (2004 μs), the new crack led to another stress jump in the curve, but no significant influence was observed. Therefore, no through-thickness tensile stress existed to promote the delamination propagation.

$\tau_{23}$ increased monotonically with the distance in the Inner Zone, reaching a peak value adjacent to Crack A’ at 1071 μs. In the Outer Zone, it decreased gradually. At this moment, the stress jump of $\tau_{23}$ was also small. Later, $\tau_{23}$ increased on a noticeable scale (1528 μs). The magnitude of the stress jump at Crack A’ was also enlarged by the crack propagation. A crest, which was about 60 MPa, appeared in the Outer Zone. After the delamination initiation (1652 μs), the crest kept moving outwards, promoting the delamination propagating. In the Inner Zone, $\tau_{23}$ reduced on a large scale, suppressing the delamination in the Inner Zone. These tendencies remained unchanged, even after the initiation of Crack B’ (2004 μs). 

In general, Crack A’ initiated the MDR and promoted its propagation in the Outer Zone by the crest of $\tau_{23}$. In the Inner Zone, Crack A’ suppressed the delamination by the stress release and formed the bottom line of the MDR. Since the location of Crack A’ was determined by the stress distribution near the impact location, “c”, i.e., the distance between two lobes was only dependent on the contact between the impactor and laminate during LVI instead of the impact energy. In general, the undelaminated region between the two parts of delamination exists at each interface of an impacted laminate [51,52]. Since this region plays an important role in CAI, it is essential to predict “c” accurately in FE models.

#### 4.2.3. Artificial Delamination

**Figure 16:**
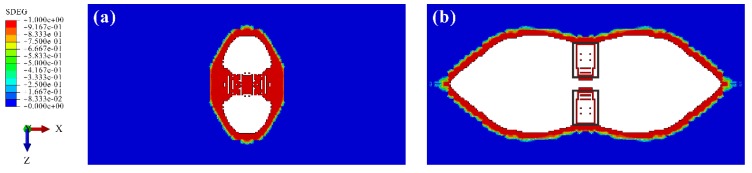
Artificial delamination: (**a**) at the upper interface; (**b**) near the NDR at the lower interface (in the black boxes).

In FE models, artificial delamination existed at the upper interface and near the NDR at the lower interface (see Figure 16). It was irrelevant to the matrix cracking and only induced by the limitations of cohesive elements.

As mentioned above, only three stress components ($\sigma_{33}$, $\tau_{13}$, and $\tau_{23}$) are computed in a cohesive element. Due to the absence of the in-plane normal stress, the cohesive elements near the transverse symmetric plane resisted the bending deformation of the plies only by the shear stress. Consequently, the shear stress was overestimated. 

Furthermore, the through-thickness compression was believed to delay the delamination [53,54,55], which was not considered in the cohesive elements in ABAQUS. According to the theories proposed by Fiedler et al. [56,57,58,59], the high-level through-thickness compressive stress delayed the local plastic deformation inside the resin-rich layer between two plies, i.e., the interlaminar interface, by increasing hydrostatic pressure. In the crack tip, the micro-cracking caused by the local plastic deformation was suppressed. Therefore, the delamination and matrix crack when its tip was near the impact location were possibly hindered by compression. 

Consequently, the cohesive elements near the transverse symmetric plane failed due to the overestimated shear stress, although their failure was supposed to be suppressed by the high-level compression induced by the impactor and stress release caused by Crack A’.

## 5. Conclusions

In this investigation, the relationship between matrix cracking and delamination in cross-ply laminates during LVI has been studied. Several experimental methods, including C-scan, micrograph, and visual inspection, were adopted to characterize the impact damage. XFEM-based FE models were established to analyze the damage mechanisms. The main conclusions are summarized as follows:

1. The tensile crack is induced by $\sigma_{22}$, while the shear cracks are mainly caused by $\sigma_{22}$ and $\tau_{23}$.
2. The tensile crack determines the outline and sizes of delamination by the stress concentration. In addition, the stress release by the opening of the shear cracks forms the two-lobe morphology of delamination.
3. The effect of the tensile crack indicates that the delamination extension is related to the global bending stiffness of a laminate.
4. The undelaminated region is only dependent on the impactor geometry and the local stiffness of a laminate, since the distance between two lobes is determined by the contact between the impactor and laminate.

Overall, the stress redistribution effect caused by CODs of the matrix cracks determines the shapes and sizes of the delamination.

Finally, this study not only provides an effective approach for impact damage modeling but also highlights the significance of modeling the shear cracks accurately since they determine the undelaminated region, which plays an important role in CAI behaviors. Based on the damage mechanisms of this study, more accurate FE models will be established in future work to analyze the impact damage of realistic stacking sequences used in aeronautical engineering. Moreover, a further study is needed to investigate the effect of the through-thickness compression on the impact damage.

## Figures and Tables

**Table 1:** Material properties of IMA/M21.

Properties		Values
Intralaminar	Elastic modulus (GPa)	$E_{11}=154$, $E_{22}=E_{33}=8.50$, $G_{12}=G_{13}=4.20$, $G_{23}=2.76$
	Poisson’s ratio	$\nu_{12}=\nu_{13}=0.35$, $\nu_{23}=0.43$
	Fracture resistance (MPa)	$R_{⊥}^{\left(+\right)A}=55.0$, $R_{⊥||}^{A}=105.0$, $R_{⊥⊥}^{A}=109.6$
	Inclination parameter	$p_{⊥⊥}^{\left(+\right)}=0.30$, $p_{⊥⊥}^{\left(-\right)}=0.30$, $p_{⊥||}^{\left(+\right)}=0.35$, $p_{⊥||}^{\left(-\right)}=0.30$
	Critical energy release rate of matrix cracking (N/mm)	$G_{Ic}=0.30$, $G_{IIc}=0.60$
Interlaminar	Strength (MPa)	N=55.0, S=68.0
	Critical energy release rate (N/mm)	$G_{Ic}=0.30$, $G_{IIc}=0.60$

**Table 2:** Delamination sizes for three impact energy levels (unit: mm): average sizes and coefficients of variation of experimental results, sizes of numerical results.

	4 J		8 J		12 J	
	Experiments	Simulations	Experiments	Simulations	Experiments	Simulations
a	8.16 (8.89%)	10.2	14.95 (6.95%)	14.2	17.59 (2.29%)	15.2
b	2.44 (10.26%)	1.0	4.48 (13.32%)	1.0	5.48 (16.36%)	1.0
c	2.92 (11.21%)	2.8	2.38 (7.56%)	2.8	2.54 (20.81%)	2.8
d	8.07 (7.33%)	10.0	12.60 (5.57%)	14.6	14.97 (5.09%)	14.8
e	23.08 (7.62%)	24.0	41.91 (8.01%)	39.6	49.60 (5.54%)	46.0
